# An online hybrid BCI combining SSVEP and EOG-based eye movements

**DOI:** 10.3389/fnhum.2023.1103935

**Published:** 2023-02-16

**Authors:** Jun Zhang, Shouwei Gao, Kang Zhou, Yi Cheng, Shujun Mao

**Affiliations:** School of Mechanical and Electrical Engineering and Automation, Shanghai University, Shanghai, China

**Keywords:** hybrid brain-computer interface (hBCI), steady-state visual evoked potential (SSVEP), electrooculography (EOG), eye movements, information transmission rate

## Abstract

Hybrid brain-computer interface (hBCI) refers to a system composed of a single-modality BCI and another system. In this paper, we propose an online hybrid BCI combining steady-state visual evoked potential (SSVEP) and eye movements to improve the performance of BCI systems. Twenty buttons corresponding to 20 characters are evenly distributed in the five regions of the GUI and flash at the same time to arouse SSVEP. At the end of the flash, the buttons in the four regions move in different directions, and the subject continues to stare at the target with eyes to generate the corresponding eye movements. The CCA method and FBCCA method were used to detect SSVEP, and the electrooculography (EOG) waveform was used to detect eye movements. Based on the EOG features, this paper proposes a decision-making method based on SSVEP and EOG, which can further improve the performance of the hybrid BCI system. Ten healthy students took part in our experiment, and the average accuracy and information transfer rate of the system were 94.75% and 108.63 bits/min, respectively.

## 1. Introduction

Brain-computer interface (BCI) technology is a new human-computer interaction technology that converts neural activities generated by brain activity into control signals, and uses these signals to control external output devices (McFarland and Wolpaw, 2011). BCI systems are divided into implantable and non-implantable types according to the acquisition method of brain signals (Birbaumer et al., 2006). Currently, the most common methods for extracting brain signals are non-implantable, including electroencephalography (EEG), functional magnetic resonance imaging (fMRI; Yoo et al., 2004), magnetoencephalography (MEG; Mellinger et al., 2007), and functional near-infrared spectroscopy (fNIRS; Hong et al., 2015). Among non-implantable BCIs, EEG is widely used for its high temporal resolution, ease of acquisition, and cost-effectiveness compared to other brain activity monitoring modalities. Electrophysiological sources in the non-implanted brain include event-related synchronization/desynchronization (ERS/ERD; Pfurtscheller, 2001), visual evoked potential (VEP; Bin et al., 2011), steady-state visual evoked potential (SSVEP), slow cortical potential (SCP; Mensh et al., 2004), μ and β rhythms (McFarland et al., 2006), and P300 evoked potentials (Gu et al., 2019).

Compared with other types of BCI systems, the SSVEP-BCI system has unparalleled advantages in real-time control and practical application. Firstly, the SSVEP-BCI system requires little or no training, whereas the P300-BCI and MI-BCI systems require a longer period of training for the individual prior to the experiment. Secondly, SSVEP is a physical response of the primary visual cortex to visual stimulation, and its signals are mainly concentrated in the occipital region of the brain and have a distinct periodic and rhythmic. Finally, the SSVEP-BCI system has a high ITR compared to other BCI systems (Li et al., 2021).

Current single-modality brain-computer interfaces face some challenges (Ma et al., 2017), including poor robustness for long-time operations, poor human-machine adaptation and system stability. In addition, the number of tasks achievable by a single-modality BCI system is limited, which restricts the ability of external output devices to accomplish complex tasks. The increase in the number of functional instructions leads to a decrease in classification accuracy, and it is difficult for single-modality BCI systems to obtain better results in practical applications. In view of the above-mentioned problems of single-modality BCI systems, the concept of hybrid brain-computer interfaces has been proposed in recent years (Chai et al., 2020; Zhu et al., 2020). A hybrid BCI is a system that mixes a single-modality BCI with another system (BCI or non-BCI system; Pfurtscheller et al., 2010; Duan et al., 2015). Hybrid BCI has multiple input modes, and the input signals can be processed in parallel or serial (Allison et al., 2011). SSVEP can form a hybrid BCI with other EEG signals, such as P300 (Panicker et al., 2011; Yin et al., 2015), electrooculography (EOG; Saravanakumar and Reddy, 2019; Zhou et al., 2020), and electromyography (EMG; Lin et al., 2016; Rezeika et al., 2018). Yin et al. proposed a hybrid BCI speller by superimposing SSVEP stimulus on P300 stimulus to increase the difference between targets in the same row or column, and then recogniz the target by a fusion method with maximum-probability estimation (MPE; Yin et al., 2015). Lin et al. proposed a hybrid BCI speller based on EMG and SSVEP, with a total of 60 targets composed by four identical sets of frequencies (Lin et al., 2016). EMG is used to identify the group, and SSVEP is used to select the targets within the group based on the flash frequency. This paradigm improves accuracy and ITR with the addition of a second selection task. A hybrid paradigm based on EOG and SSVEP was proposed by Saravanakumar et al. In such paradigms, EOG is used for selecting regions or groups by blinking, while SSVEP is used for recognizing targets (Saravanakumar and Reddy, 2019). This SSVEP-EOG paradigm requires mental resources for the blink selection of groups, which is prone to fatigue, and the eye-movement features in EOG are not fully utilized.

This paper proposes a new method to improve the hybrid BCI performance by combining SSVEP and EOG-based eye movement. A total of 20 characters are distributed in five regions of the GUI and start flashing at different frequencies and initial phases simultaneously. At the end of the flashing, the characters in four regions move in different directions, and the user continues to follow the target through eye movements. In this paper, both the CCA and FBCCA methods were used to detect SSVEP, and it was found that the FBCCA method has more performance. The eye movements are judged by analyzing the EOG waveform features, and the 20 target grouping situations are decided based on the eye movement. Based on the online hybrid BCI system based on SSVEP and EOG-based eye movement proposed in this paper, ten healthy school students participated in our experiments. This system has satisfactory performance in the experiment with an average ITR of 108.63 bits/min and an average accuracy of 94.75%.

## 2. Materials and methods

### 2.1. Data acquisition

**Figure 1:**
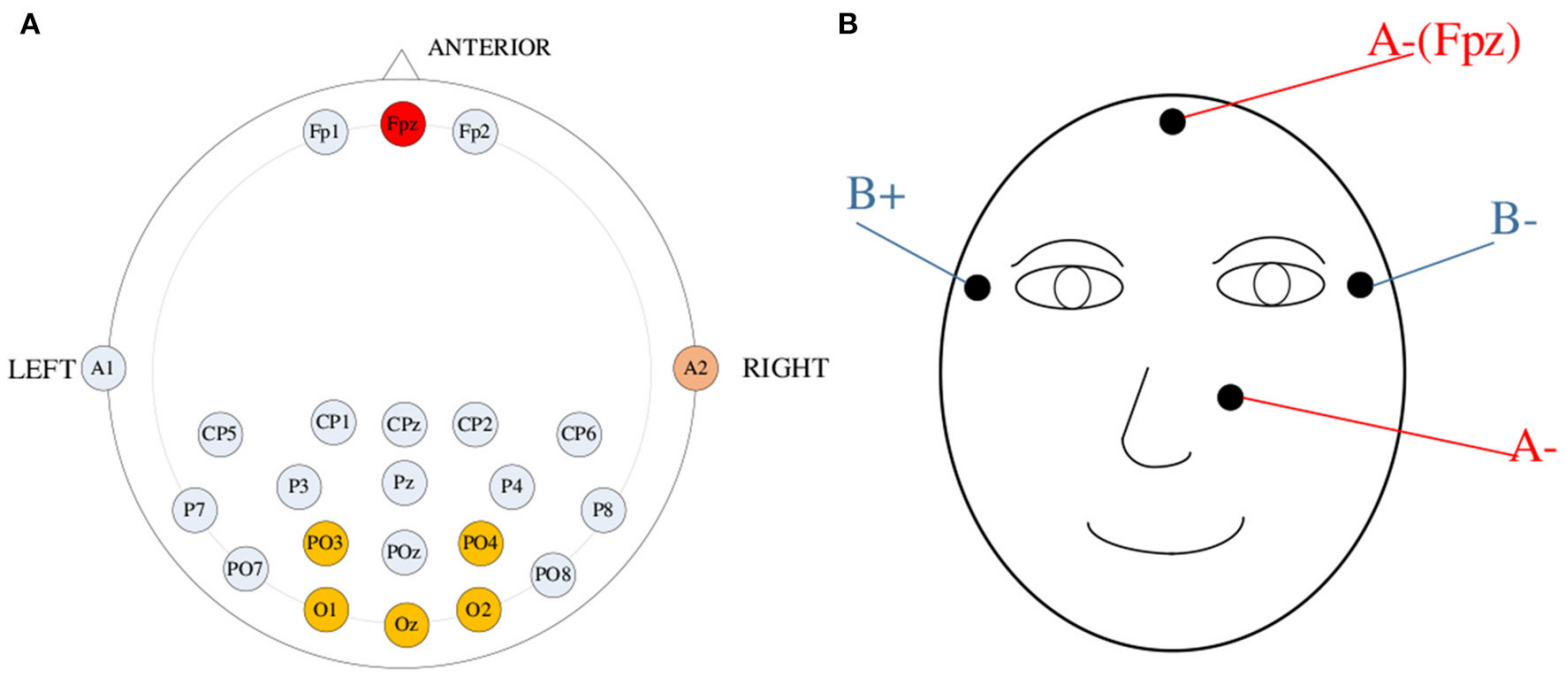
**(A)** Location of electrodes for EEG signals acquisition. **(B)** Location of electrodes for EOG signals acquisition.

Based on the ADS1299 chip from Texas Instruments, we designed an eight-channel high-precision signal amplifier with a sampling rate of 250 Hz. The frequency passband of the amplifier is from 0.15 Hz to 200 Hz for simultaneous acquisition of EEG and EOG. Based on the standard position in the 10–20 system, the forehead (Fpz) and left mastoid electrode (A1) were selected as the reference electrode and right leg drive electrode (Figure 1A), respectively, and five channels (PO3, PO4, Oz, O1, and O2) in the occipital region were selected to acquire EEG data. In this paper, a bipolar lead was used to acquire EOG data, and the acquisition electrodes were placed as shown in Figure 1B, with four electrodes placed on the vertical and horizontal axes of the eye for measuring eye movements. The ground electrode for EEG signal acquisition was used as the positive electrode for the vertical EOG signals.

### 2.2. Stimulation paradigm

**Figure 2:**
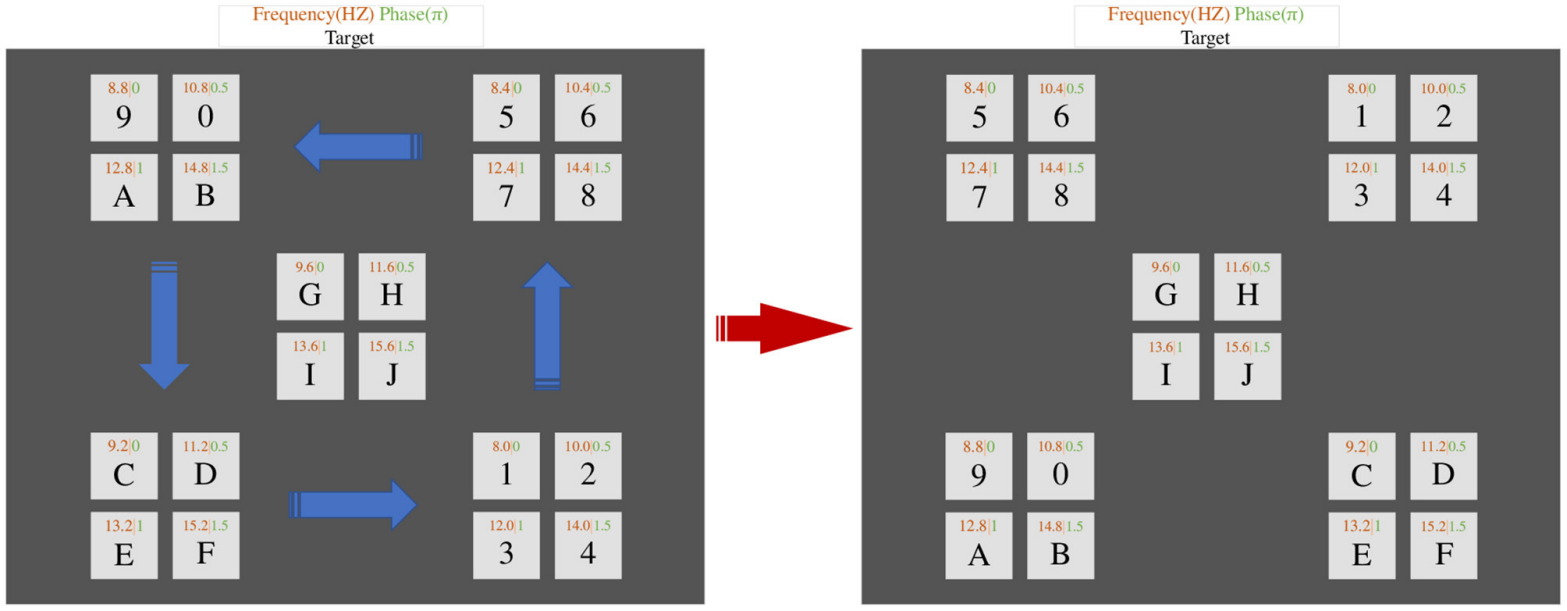
Schematic diagram of the button and changes layout displayed on the GUI. The blue arrows indicate the direction of movement of the regions, and the frequency and initial phase of each target flicker used for SSVEP are also shown in the diagram. The frequency interval in the same region is 2 Hz and the phase interval is 0.5π.

Sinusoidal stimulation can not only effectively solve the problem of screen refresh rate limitation but also alleviate the problem of experimental fatigue in SSVEP (Jia et al., 2010). Furthermore, the phase information can enhance the discrimination of SSVEP at similar frequencies. Based on the above advantages, this paper adopts a periodic sinusoidal visual stimulation paradigm combining frequency and phase information to induce SSVEP (Chen et al., 2014, 2015b). As shown in Figure 2, the interface has a total of 20 buttons, and the interface is divided into five regions (up, down, left, right, and middle), and four buttons are evenly distributed in each region. Each button flashes at a different frequency (8–15.8Hz in 0.4 Hz intervals) and phase (0–1.5π in 0.5π intervals). The sinusoidal stimulus sequence Sti(n) used to induce SSVEP is as follows:


(1) $$\begin{array}{l} \text{Sti}(n)=\frac{1}{2}(1+\sin(2\pi f_{\left(m,k\right)}(n/FPS)+\theta_{\left(m,k\right)}))\\ f_{\left(m,k\right)}=[f_{0}+(m-1)\Delta f_{1}\text{+}(k-1)\Delta f_{2}],m=1,2,3,4,5\\ \theta_{\left(m,k\right)}=[\theta_{0}+(k-1)\Delta \theta ],\text{k=1},\text{2},\text{3},\text{4} \end{array}$$

The stimulus sequence Sti(n) is from 0 to 1 (0 is black and 1 is white), which is modulated by frequency and phase, and FPS is the screen refresh rate. Where m is the index of the interface region, and Δ*f*_1_ (0.5 Hz) is the frequency interval of different regions; *k* is the index of the target in the same region; Δ*f*_2_ (2 Hz) is the frequency interval, and Δθ (0.5 π) is the phase interval of the same region.

Specifically, the designed paradigm of this paper includes two stages: the SSVEP stimulus stage and the eye movement stimulus stage. In the first stage of the SSVEP stimulus, 20 buttons start flashing simultaneously (for 1.1 s), and the subject stares at the button of his/her choice. The second stage is the eye movement stimulus. When the 1.1S sinusoidal stimulation ended, 16 buttons in four regions (top left, bottom left, top right, and bottom right) in the GUI will move in different directions. When the button starts moving, the subject continues to follow the target with his/her eyes, and the corresponding eye movement occurs. As shown in Figure 2, the left GUI shows the initial position at the beginning of the button movement, and the right GUI shows the position at the end of the button movement. The blue arrow indicates the direction of button movement. Each button moves at a constant speed, gradually moving from the initial position to the target position, with a total time of 0.3 s. The button stays for 0.2 s after reaching the target position. When the eye movement stimulus stage is completed, the position of each button is suddenly reset back to its initial position, which ensures that the initial position of each button remains the same. At the end of a single experiment the border color change of the characters analyzed by the system turns red for 1S. The method of using 20 buttons divided into five regions evenly distributed so that the frequency gap between two buttons in the same region is 2 Hz, and the phases are not the same. The design of button movement in the GUI can induce eye movements and group the buttons by analyzing the EOG signals.

**Figure 3:**
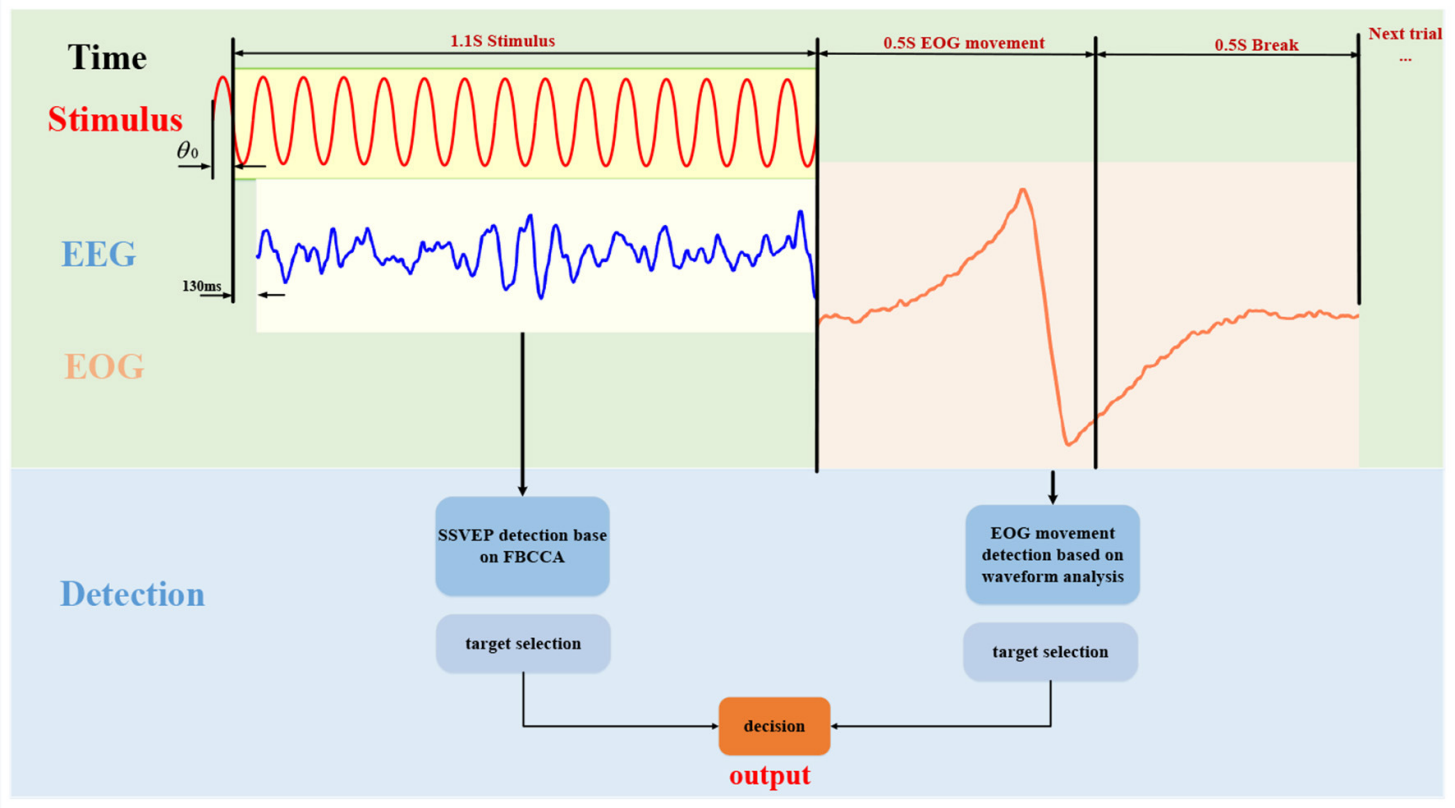
The online experiment process of the hybrid BCI system proposed in this paper. Firstly, 1.1 s of EEG data is acquired for SSVEP detection, and then 1s of EOG data (including 0.5 s of rest time EOG signals) is acquired for eye movement direction detection. A decision is made based on the results of the two detections, and the final output is obtained.

Figure 3 illustrates the stimulus process during a representative trial of the online experiment, as well as an example of EEG and EOG signals processing and final decision method. Considering that the visual system has a response delay, the EEG data for the first 130 ms are discarded during SSVEP analysis (Di Russo and Spinelli, 1999). The eye movement signals will have obvious peaks or valleys when the eye movement is stimulated. Experiments have found that some peaks or valleys disappearing tails may occur during rest periods. In order to improve the accuracy of eye movement analysis, we collect EOG signals during stimulation time and rest time to analyze eye movement direction comprehensively. Specifically, each trial consisted of a 1.6 s stimulus and a 0.5 s rest period (the time from the end of a trial stimulus to the start of the next trial). The following paper will describe the signal processing and decision-making process in detail.

### 2.3. EEG signal processing

CCA is a multivariate statistical method that is used to analyze the correlation that exists between two sets of multidimensional variables. The CCA method can be used for SSVEP detection in multichannel EEG signals (Lin et al., 2006). Compared with the CCA method, the FBCCA method uses harmonic information more effectively when detecting SSVEP (Chen et al., 2015a). The FBCCA method consists of three main steps. Firstly, the input EEG data is decomposed into *N* sub-band components by a band-pass filter set. Then the CCA method is performed on each of the *N* sub-band components. Finally, the correlation coefficients of the *N* sub-band components are weighted and averaged to obtain the overall correlation coefficient value of each stimulus frequency, and the largest correlation coefficient value corresponding to the frequency is selected as the final recognition result. In this study, this paper evaluates the performance of both CCA and FBCCA methods for SSVEP identification. In this study, the performance of CCA and FBCCA methods for SSVEP recognition in online experiments is first compared, and then the offline analysis compares the classification results of FBCCA and CCA methods with different window lengths. In this paper, harmonic components of *N* sub-bands (*N* = 3 in this study) are extracted from the EEG signals X using Butterworth infinite impulse response (IIR) bandpass filters. These sub-band filters have the same upper bound frequency (77 Hz) but different lower bound frequencies. For the nth sub-band component *X*_*n*_, the lower bound frequency is *n*×7 Hz.

### 2.4. EOG signals processing

**Figure 4:**
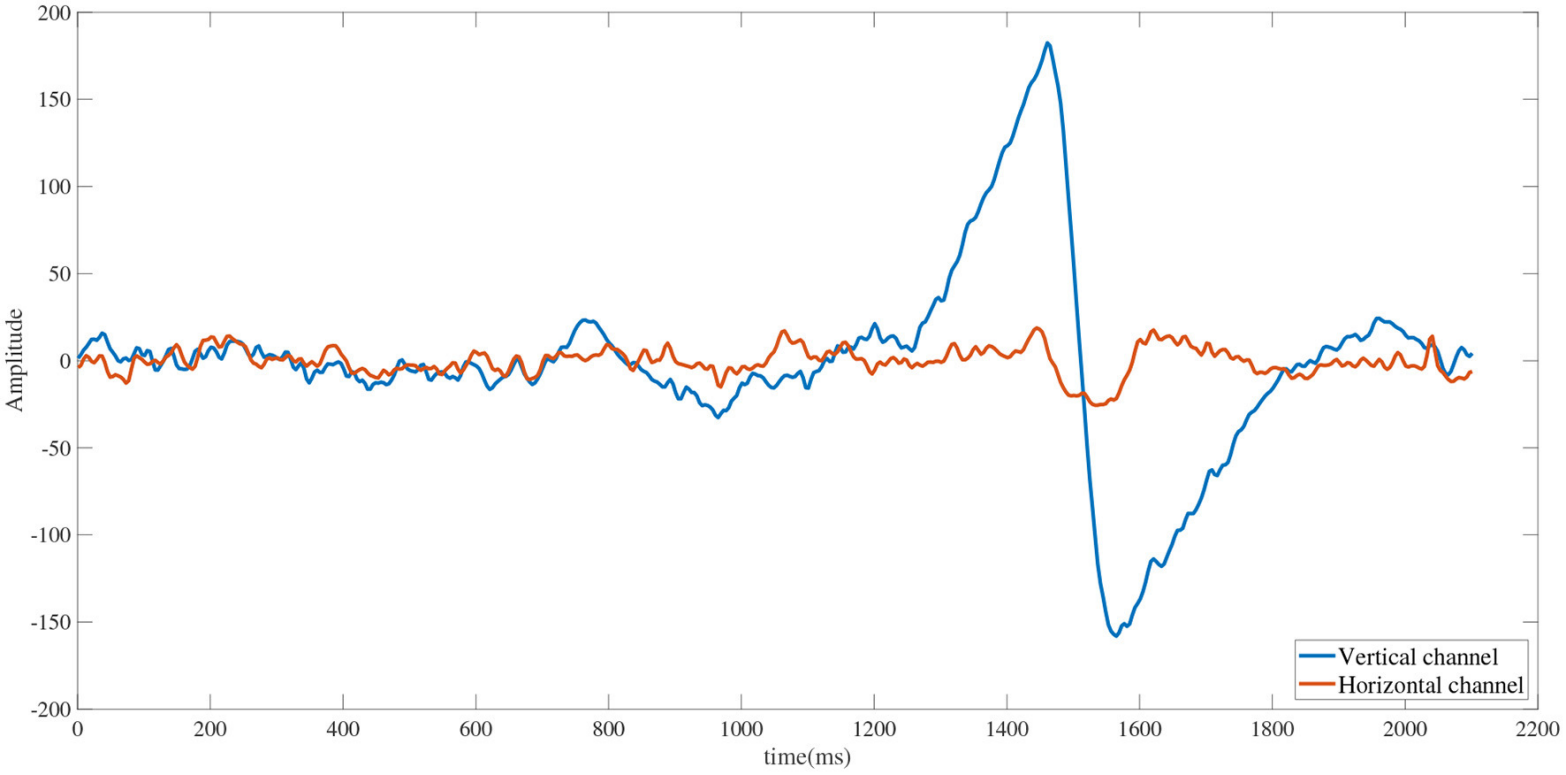
Waveforms of horizontal and vertical channels during eye movements (left). Blue line indicates horizontal channels and orange line indicates horizontal channels.

At the end of the SSVEP stimulus, the buttons in the four regions will move, and the subjects continue to follow the selected target with their eyes, and the corresponding eye movements occur. The EOG signals were recorded for 1S (250 sampling points) segments after the end of the SSVEP stimulus, and the EOG signals were filtered to the 1–10 Hz range using a 3rd-order Butterworth bandpass filter. As shown in Figure 4, the amplitude change of the channel signal corresponding to eye movement is greater than the amplitude variation of the channel signal corresponding to no eye movement (Barea et al., 2012). The root mean square (RMS) of the EOG vertical channel and horizontal channel was calculated by the following formula to determine which channel has eye movement:


(2) $$H_{rms}=\;\sqrt{\frac{\sum_{i=1}^{N}x^{2}{}_{i}}{N}},V_{rms}=\;\sqrt{\frac{\sum_{i=1}^{N}x^{2}{}_{i}}{N}}$$

**Figure 5:**
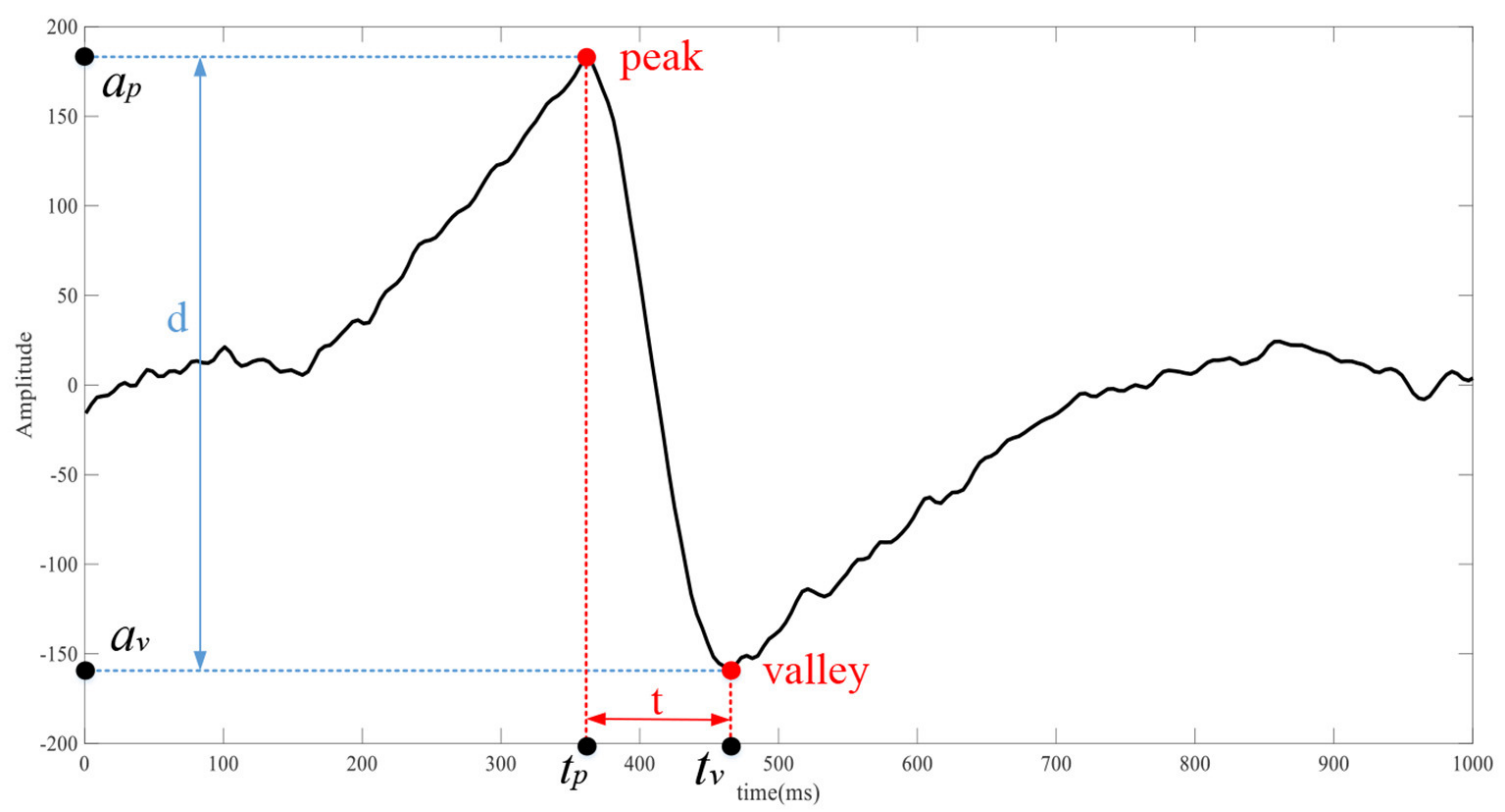
The waveform of the corresponding channel of eye movement, *d* is the distance between the peak and the valley, and *t* is the time difference between the peak and the valley.

*H*_*rms*_ is the RMS of the EOG horizontal channel; *V*_*rms*_ is the RMS of the EOG vertical channel; *x*_*i*_ is the amplitude of the EOG signals at the i-th sampling point of the corresponding channel; *N* are the number of sampling points. As shown in Figure 5, the EOG signals in the channel corresponding to the eye movement signal has obvious time-domain waveform features. The waveform features of EOG are calculated by the following formula:


(3) $$\begin{array}{l} \begin{array}{c} H_{d}=a_{p}-a_{v},H_{t}=t_{p}-t_{v}\\ V_{d}=a_{p}-a_{v},V_{t}=t_{p}-t_{v} \end{array} \end{array}$$

*H*_*d*_ and *V*_*d*_ is the peak-to-peak values of the channels. *H*_*t*_ and *V*_*t*_ is the time of the peak minus the time of the trough of the channel.

**Figure 6:**
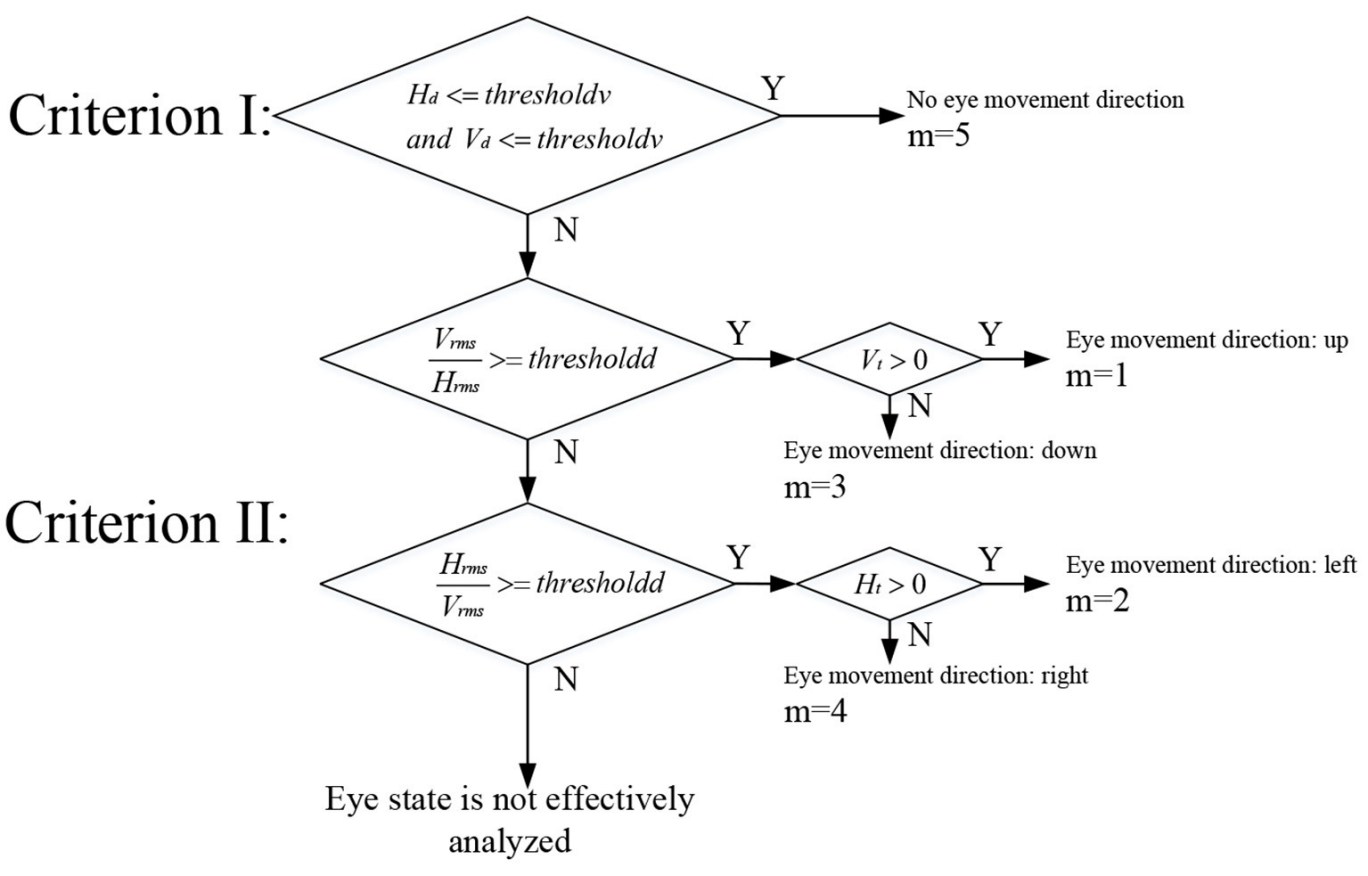
Flow chart of eye movement recognition.

The following three Criterions were used to make decisions about eye movement direction (Figure 6).

Criterion I: *H*_*d*_ < = *thresholdv* *and* *V*_*d*_ < = *thresholdv*, *thresholdv* is the threshold value of the peak-to-peak value.

Criterion II: $\frac{H_{rms}}{V_{rms}}>=thresholdd\text{ or }\frac{Vrms}{Hrms}>=thresholdd$, *thresholdd* is the threshold value of the channel RMS difference.

As shown in Figure 6, if Criterion I is satisfied, no eye movement occurs, and the subject stares at the middle four buttons (the value of the index m of the interface region is 5). If Criterion I is not satisfied, continue to analyze the direction of eye movement by Criterion II. If Criterion II is satisfied, continue to judge the specific eye movement direction through *H*_*t*_ or *V*_*t*_. If both Criterion I and Criterion II are not satisfied, the eye state cannot be determined.

### 2.5. Decision-making method based on SSVEP and EOG

**Figure 7:**
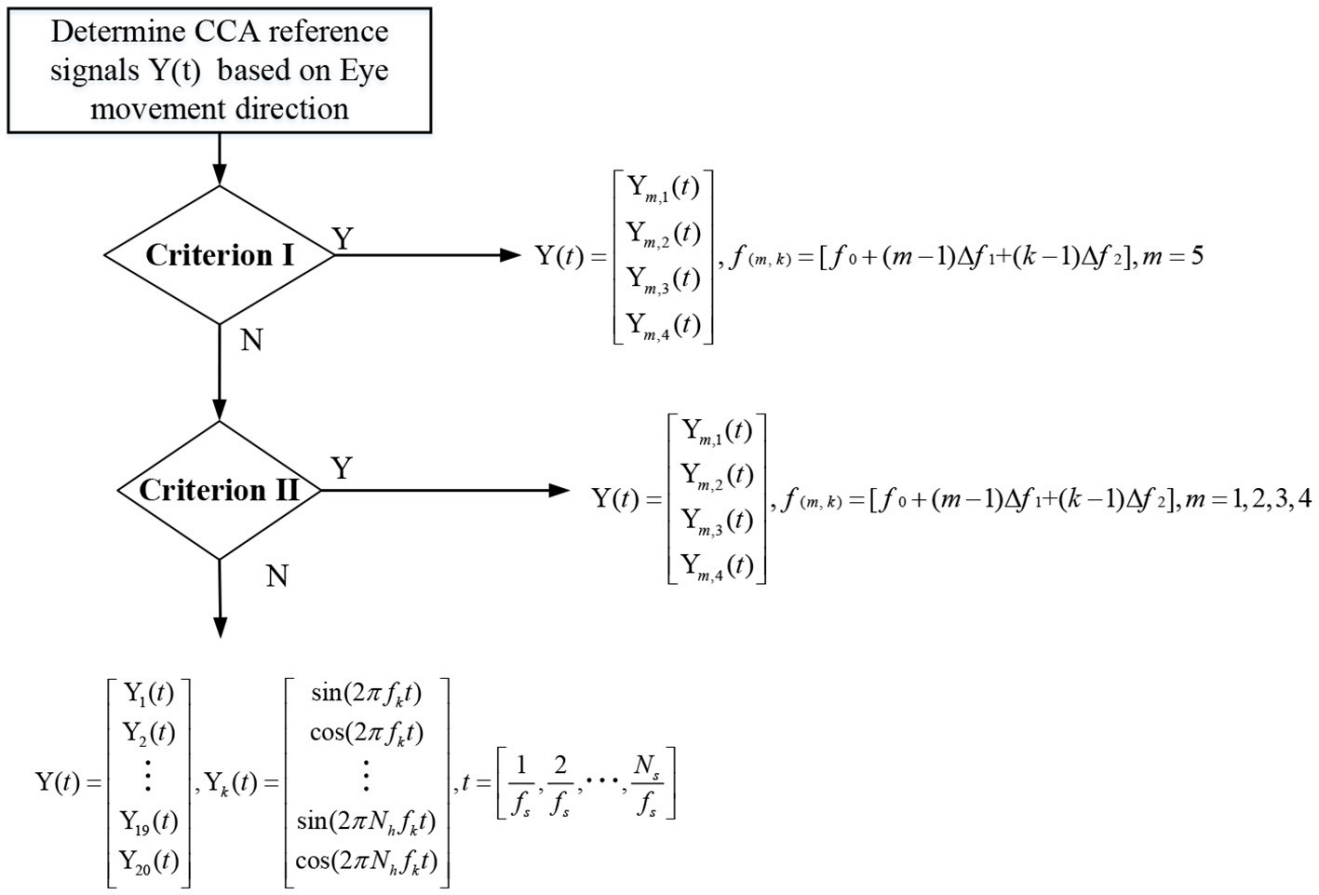
Flow chart of decision-making methods.

This paper evaluates the online performance of SSVEP through CCA and FBCCA methods, and analyzes whether the eye move and the direction of movement through waveform analysis. Decisions on the final output were made using the following methods (Figure 7).

Case 1: When the eye movement features satisfy Criterion I or Criterion II, the eye state is effectively analyzed. The 20 stimulus targets were grouped and selected according to the results of no eye movement or the direction of specific eye movement. The template signal of CCA was composed of four stimulus frequencies of the grouped results.

Case 2: None of the eye movement features satisfies Criterion I or Criterion II, indicating that the eye state is not effectively analyzed. The template signals composed of all stimulus frequencies were directly selected for CCA analysis and FBCCA analysis.

## 3. Experiments and results

We recruited 10 students (seven boys and three girls) from the school to participate in the experiment. The following online spelling experiment was conducted in a quiet environment, and the results were analyzed after completion of the experiment.

### 3.1. Spelling test experiment

The spelling system designed in this paper consisted of a laptop computer with an extended display, and the designed visual stimulation was presented on the extended display with a screen refresh rate of 60 Hz. Subjects were asked to sit in front of the laptop display, with the distance between the display and the subject kept at about 40 cm.

In this experiment, we need to test the online performance of the hybrid BCI system based on SSVEP and EOG-base eye movement proposed in this paper. Before each stimulus, the background color of the character to be selected changes to red at break time (0.5 s), which is the symbol to indicate which button is the target button. The order of selection is from the character "1" to "j." Each experiment contains six sets of stimuli, each requiring the subject to select each character in a specified order. He/she was required to gaze at the flashing character and then complete the specified eye-movement actions according to the GUI cues. In a complete trial, each character on the screen was selected six times (120 pre-specified characters).

### 3.2. Analysis of results

We choose to use accuracy, ITR (bits/min) to evaluate the performance of the system. The ITR represents the amount of information output by the system per unit time and is calculated as follows:


(4) $$\begin{array}{l} ITR=\frac{60\left(\log_{2}M+P\log_{2}P+\left(1-P\right)\log_{2}\left(\frac{1-P}{M-1}\right)\right)}{T} \end{array}$$

Where M is the number of characters. P is the accuracy, and T is the average response time. Each experiment included 1.6 s stimulation and 0.5 s rest time, so T was fixed at 2.1 s.

**Figure 8:**
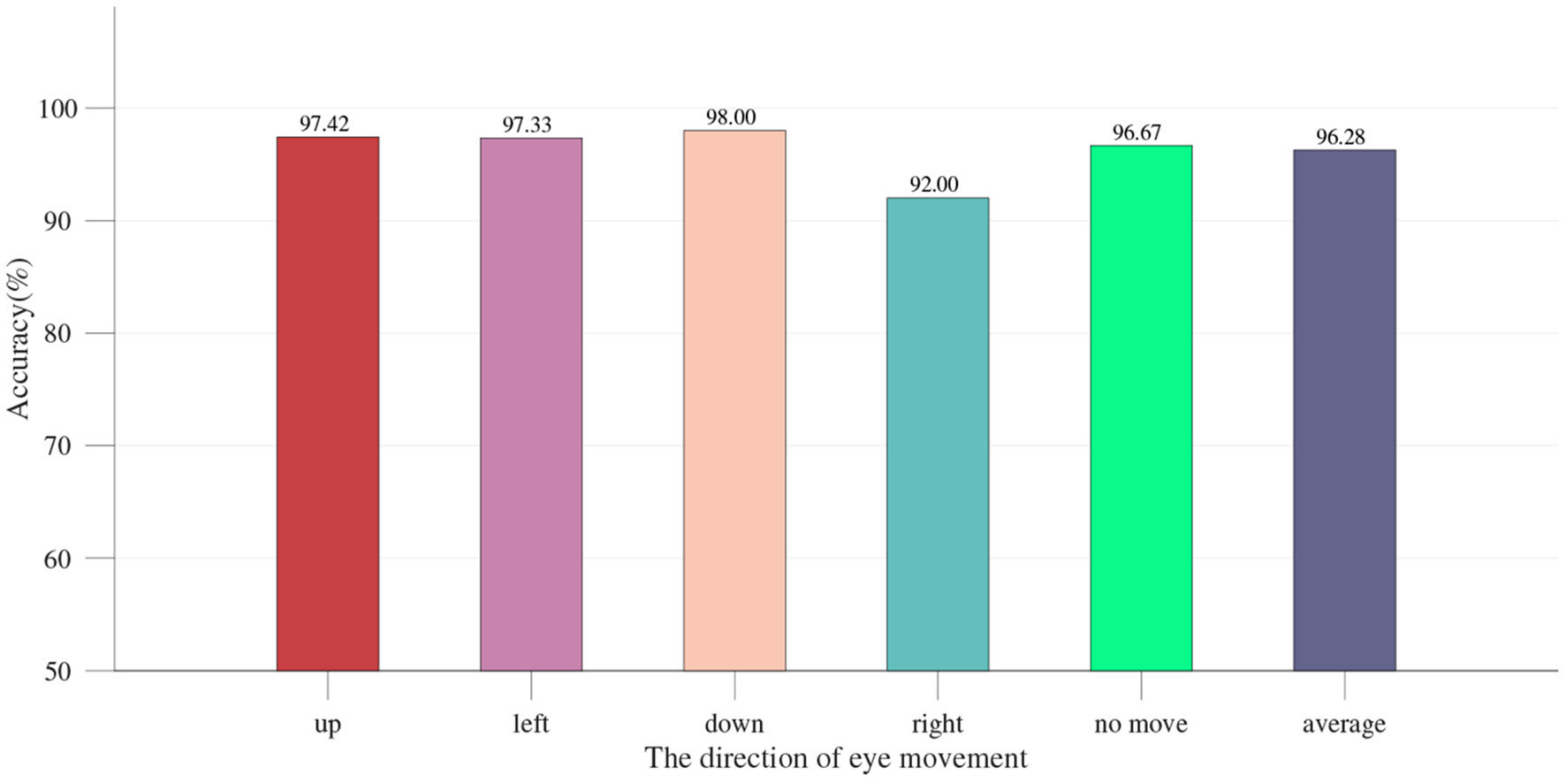
Eye movement classification accuracy of dual-lead channel EOG electrodes.

**Figure 9:**
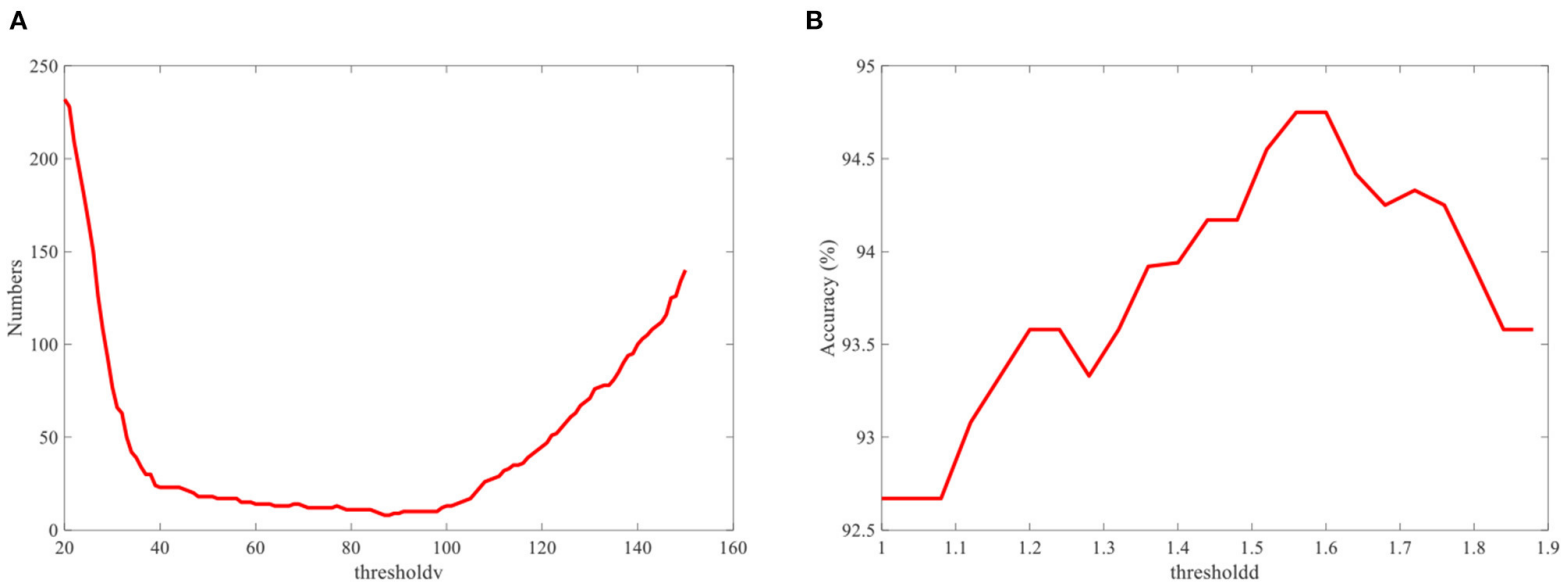
**(A)** Relationship between the number of misjudgments and the *thresholdv*. **(B)** Relationship between the classification accuracy of the hybrid system and the *thresholdd*.

**Figure 10:**
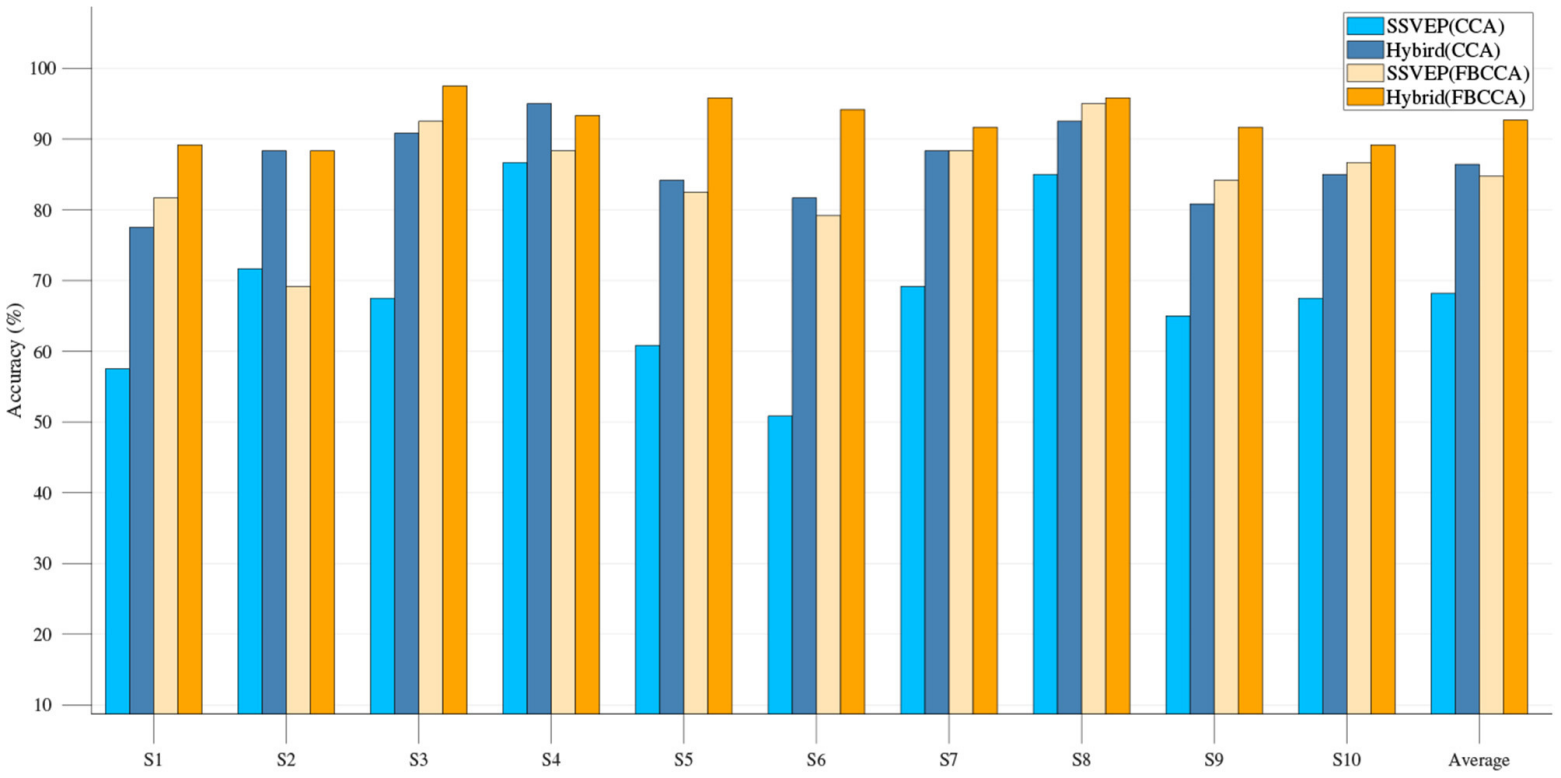
Classification accuracy of single-modality SSVEP and hybrid BCI system based on CCA and FBCCA.

The single-modality part is first analyzed, including the results of the EOG-based eye movement classification accuracy and the SSVEP classification accuracy for each experiment. In EOG eye movement recognition, the *thresholdv* is used to determine whether eye movements occur. We analyze the relationship between the number of incorrect judgments and the *thresholdv*. As shown in Figure 9A, the number of misjudgments first decreases and then increases as *thresholdv* increases. The role of the *thresholdd* is to determine the direction of eye movements. When the value of *thresholdd* is 1, corresponding to a simple hybrid system, the EOG signal must analyze the direction of eye movements to determine the region of the button. Figure 8 shows the recognition accuracy of EOG eye movements at this time, with results of 97.42, 97.33, 98.00, 92.00, and 96.67% (mean 96.28%). When the value of *thresholdd* is >1, the hybrid system uses the decision-making method based on SSVEP and EOG proposed in the previous section (Figure 7). The region of the button is only confirmed when the eye state is effectively analyzed. We analyze the relationship between the classification accuracy of the hybrid system and the *thresholdd*. As shown in Figure 9B, the classification accuracy of system first increases and then decrease as *thresholdd* increases. Based on the results of the above analysis, the value of *thresholdv* chosen in this paper is 84 and the value of *thresholdd* is 1.56. Figure 10 shows the SSVEP classification accuracy based on different methods.The above results show that each eye movement direction of the EOG and each frequency of the SSVEP can be well-identified, confirming the validity of the single modality identification parameter selection.

**Figure 11:**
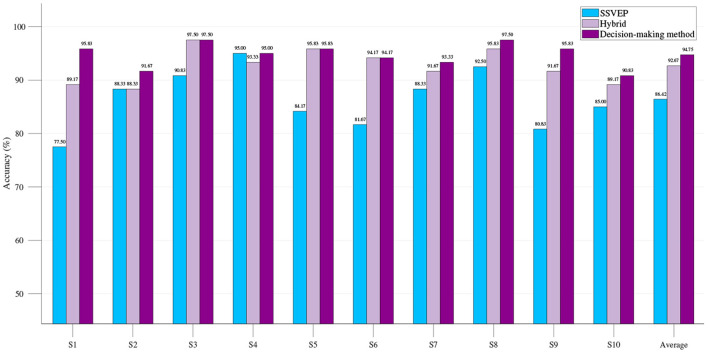
Classification accuracy of single-modality SSVEP BCI system, hybrid BCI system, and hybrid BCI system using decision-making method based on SSVEP and EOG.

After EEG combined with EOG to form a hybrid BCI, compared with the single-modality BCI, the classification accuracy of each group was greatly improved (Figure 10). For those subjects with relatively low eye movement classification accuracy (< 94%), the classification accuracy can be further improved by using the decision-making method based on SSVEP and EOG proposed in this paper (Figure 11). The hybrid BCI system corresponds to the case where the value of *thresholdd* is 1. The hybrid BCI system using decision-making method based on SSVEP and EOG corresponds to the case where the value of *thresholdd* is 1.56. On average, all subjects performed satisfactorily with high accuracy of 94.75 ± 3.92% and relatively high ITR of 108.63 ± 8.91 bits/min.

## 4. Discussion

A hybrid BCI system combining SSVEP and EOG signals is proposed in this paper. SSVEP is induced by JFPM [16], where the phase encoding is merged into the frequency encoding. Specifically, by modulating the initial brightness of these flashing buttons, different initial phases are exhibited in the sinusoidal stimulation, which can increase the differences between targets within the same region of the GUI. The traditional hybrid BCI method needs to consume mental resources and use the signal of another system to actively group or select targets. The GUI designed in this paper solves this drawback by making the buttons move, so the user can unconsciously make eye movements just by staring at the target without consuming mental resources. The results of eye movements were determined by waveform analysis of the acquired EOG signals. Firstly, according to the paradigm proposed in this paper, SSVEP and EOG do not overlap during signal analysis due to eye movements after SSVEP stimulus. Secondly, EOG components are concentrated in prefrontal regions, which are located away from the SSVEP, whereas SSVEP components are mainly concentrated in the occipital regions of the brain (Bin et al., 2009). Finally, according to the frequency bands characterized in this study, the EOG signals appear in the low frequency (1–5 HZ), while the SSVEP appears in the middle frequency band (8–16 HZ).

Although the number of parts of EOG-based eye movement direction recognition can be increased, the increase in the number means that the accuracy of eye movement recognition decreases. The number of SSVEP stimulus is limited by the limited screen resolution, and increasing the number also reduces accuracy. For these reasons, the hybrid paradigm designed in this paper contains four eye movement directions, each direction and no eye movement contains four buttons (20 buttons in total) flashing at different frequencies. The EOG is used to group the SSVEP in the hybrid system, and the SSVEP grouping error will obviously reduce the performance of the hybrid BCI system. In order to improve the accuracy of EOG signals grouping, the decision-making method proposed in this paper judges whether the eye state is effectively analyzed through the set threshold. The EOG only acts as a group when the eye state is effectively analyzed, otherwise the SSVEP is analyzed by selecting a template signal consisting of all stimulus frequencies. Table 1 shows the experimental results of EOG-based eye movement system, SSVEP single-modality system, and hybrid BCI system. It can be found that compared with the single-modality system, the ITR of the hybrid BCI system increases and the accurate classification rate improves, while the decision-making method based on SSVEP and EOG proposed in this paper can further improve the performance of the hybrid BCI system (Figure 11).

**Table 1:** Experimental results of online systems: accurate classification rate and ITR for EOG-only, SSVEP-only and hybrid systems.

	**EOG**	**SSVEP (based CCA)**	**Hybrid (EEG-EOG)**
**Subject ID**	**Accurate classification rate (%)/ITR (bits/min)**	**Accurate classification rate (%)/ITR (bits/min)**	**Accurate classification rate (%)/ITR (bits/min)**
S1	90.00/47.23	57.50/43.80	95.83/111.29
S2	95.00/55.30	71.67/64.53	91.67/101.55
S3	100.00/66.34	67.50/58.05	97.50/115.63
S4	95.00/55.30	86.67/91.12	95.00/109.23
S5	98.33/61.89	60.83/48.35	95.83/111.29
S6	98.33/61.89	50.83/35.24	94.17/107.24
S7	93.33/52.44	69.17/60.60	93.33/105.30
S8	96.67/55.30	85.00/87.85	97.50/115.63
S9	95.00/46.70	65.00/54.32	95.83/111.29
S10	91.67/49.76	67.50/58.05	90.83/99.73
Mean	93.70/53.05	68.17/59.07	94.75/108.63

**Figure 12:**
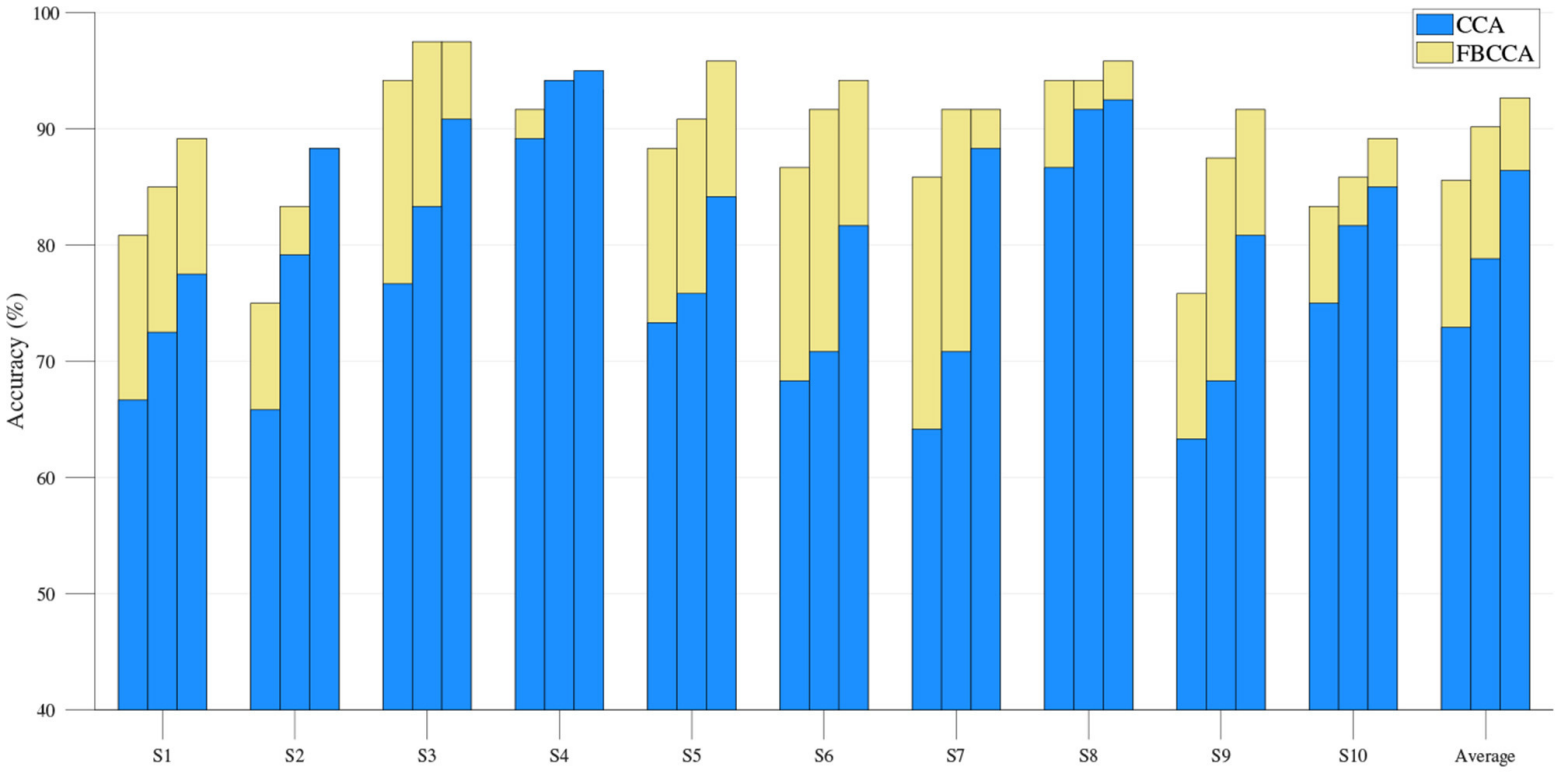
Offline classification accuracy of CCA and FBCCA methods at different window lengths. Three bars in each experiment indicated three window length from 0.8 s (left) to 1.2 s (right) at a step of 0.2 s. The blue and yellow bars show the results of the CCA and FBCCA, respectively.

Our offline analysis compares the classification results of FBCCA and CCA methods at different window lengths, as shown in Figure 12. The statistical results show that the classification accuracy of FBCCA is significantly better than the results of CCA in most experiments. In recent studies, TRCA and eCCA methods are used in the analysis of SSVEP (Wang et al., 2014; Nakanishi et al., 2015, 2017), but the work of these methods to collect SSVEP training data increases the difficulty of the system. In contrast, the FBCCA method does not require data for training, and the performance of online analysis is also high, with an average accuracy of 94.75% and an ITR of 108.63 bits/min.

Table 2 compares the performance between the hybrid BCI system proposed in this paper and the traditional hybrid BCI systems. Therefore, the ITR of the system proposed in this paper are higher than those of traditional hybrid BCI systems.

**Table 2:** Comparison between our system and several other hybrid BCI systems.

**References**	**Paradigm**	**RT(s)**	**Number of commands**	**Accuracy (%)**	**ITR (bits/min)**
Xu et al. (2014)	SSVEP + P300	≥4	36	87.80	54.00
Yin et al. (2015)	SSVEP + P300	≥5	64	95.18	50.41
Lin et al. (2016)	SSVEP + EMG	5	60	85.80	90.90
Rezeika et al. (2018)	SSVEP + EMG	≥5	30	93.75	31.05
Saravanakumar and Reddy (2019)	SSVEP + EOG	≥4	36	98.33	69.21
Proposed in this paper	SSVEP + EOG	2.1	20	94.75	108.63

## 5. Conclusion

In this study, an online hybrid BCI system based on SSVEP and EOG-based eye movements was designed. According to the eye movement direction, 20 characters are divided into five parts. EOG is used to classify a target in which part, and SSVEP is used to classify the target. The average accuracy and ITR were 94.75% and 108.63 bits/min, respectively, and higher than either of the two single-modality systems.

## Data availability statement

The raw data supporting the conclusions of this article will be made available by the authors, without undue reservation.

## Ethics statement

The studies involving human participants were reviewed and approved by Ethics Committee of Shanghai University (ECSHU). The patients/participants provided their written informed consent to participate in this study.

## Author contributions

JZ, SG, and KZ proposed the idea of the paper and designed the paradigm of the experiment. JZ designed and debugged the acquisition equipment. JZ, SM, and YC organized the experiments and analyzed the data. All authors contributed to this paper.
